# Differential expression of protein kinase C isoforms in coronary arteries of diabetic mice lacking the G-protein Gα11

**DOI:** 10.1186/1475-2840-9-93

**Published:** 2010-12-29

**Authors:** Dieter Paul Hoyer, Yüksel Korkmaz, Sabine Grönke, Klaus Addicks, Nina Wettschureck, Stefan Offermanns, Hannes Reuter

**Affiliations:** 1 Department of Internal Medicine III, University of Cologne, Kerpener Str. 62, 50937 Cologne, Germany; 2 Dept. of Operative Dentistry, Periodontics and Endodontics, Heinrich-Heine-University, Düsseldorf, Germany; 3 Insitute of Anatomy I, University of Cologne, Joseph-Stelzmann Str.9, 50931 Cologne, Germany; 4 Max-Planck-Intitute for Heart and Lung research, W.G. Kerckhoff-Institute, Parkstrasse 1, 61231 Bad Nauheim, Germany

## Abstract

**Background:**

Diabetes mellitus counts as a major risk factor for developing atherosclerosis. The activation of protein kinase C (PKC) is commonly known to take a pivotal part in the pathogenesis of atherosclerosis, though the influence of specific PKC isozymes remains unclear. There is evidence from large clinical trials suggesting excessive neurohumoral stimulation, amongst other pathways leading to PKC activation, as a central mechanism in the pathogenesis of diabetic heart disease. The present study was therefore designed to determine the role of G_q_-protein signalling via Gα_11 _in diabetes for the expression of PKC isozymes in the coronary vessels.

**Methods:**

The role of Gα_11 _in diabetes was examined in knockout mice with global deletion of Gα_11 _compared to wildtype controls. An experimental type 1-diabetes was induced in both groups by injection of streptozotocin. Expression and localization of the PKC isozymes α, βII, δ, ε, and ζ was examined by quantitative immunohistochemistry.

**Results:**

8 weeks after induction of diabetes a diminished expression of PKC **ε **was observed in wildtype animals. This alteration was not seen in Gα_11 _knockout animals, however, these mice showed a diminished expression of PKCζ. Direct comparison of wildtype and knockout control animals revealed a diminished expression of PKC δ and ε in Gα_11 _knockout animals.

**Conclusion:**

The present study shows that expression of the nPKCs δ and **ε **in coronary vessels is under control of the g-protein Gα_11_. The reduced expression of PKC ζ that we observed in coronary arteries from Gα_11_-knockout mice compared to wildtype controls upon induction of diabetes could reduce apoptosis and promote plaque stability. These findings suggest a mechanism that may in part underlie the therapeutic benefit of RAS inhibition on cardiovascular endpoints in diabetic patients.

## Background

Diabetes mellitus is a major risk factor for developing coronary artery disease. In the diabetic population atherosclerosis accounts for 80% of all deaths compared to one third in the general population. Numerous studies have addressed the complex pathogenesis of coronary artery disease. It is assumed that distinct stages can be defined in the progression of the disease. These contain the monocyte and endothelium mediated oxidation of LDL (oxLDL), the monocyte recruitment and extravasation, the formation of foam cells most likely mediated through scavenger receptors followed by inflammatory responses that lead to the building of atherosclerotic plaques, which potentially rupture and lead to myocardial infarction/ischemia.

In a diabetic environment certain mechanisms exist which are incriminated to promote the formation of atherosclerotic lesions. The building of advanced glycation end products (AGEs) which is mainly determined by the level of glucose and time of exposure, the induction of hyperglycaemia induced oxidative stress, hyperglycaemia mediated inflammation through cytokines including activation of monocytes and adipocytes, the activation of the hexosamine pathway and the regulation of proteinkinase C (PKC) activity are interacting and engaging in the pathogenesis of atherosclerosis as described above. Numerous operations of these demonstrate a participation of PKC activation. First of all PKC activation increases the pro-oxidant environment by increasing the production of reactive oxygen species in the endothelium [1] supporting the formation of oxLDL. Next the endocytosis of oxLDL can be mediated by scavenger receptors that are controlled by PKC [2]. This oxLDL leads to the release of granulocyte-macrophage-colony-stimulating-factors (GM-CSF) which can be blocked by general PKC blockage [3]. Other factors beside the GM-CSF-release contribute for the monocyte adhesion like the expression of P-selectin that is upregulated by PKC activation [4]. Adhesion of monocytes and accumulation of oxLDL results in the formation of cholesterol loaded foam cells and characteristic atherosclerotic lesions. Typical for the development of more complex lesions is the proliferation and migration of vascular smooth muscle cells (VSMCs) in the subendothelial space which is influenced by different isozymes of PKC [5]. Furthermore the expression of different metallomatrix-proteinases (MMPs), which play a crucial role for the plaque stability and the process of plaque rupture [6], are affected by PKC. So the central role of PKC in all steps of the formation of atherosclerotic plaques is reflected broadly. In addition, functional examinations show a differential pattern of endothelial barrier properties and therefore the permeability of the endothelium depending on the activation of PKC [7].

PKC is a family of at least twelve isozymes and the particular involvement of the different isozymes is only poorly understood. The family of PKC isozymes is divided in subgroups in order of their enzymatic qualities: The conventional PKCs (cPKC) consist of the isozymes α, β and γ, which are activated in the function of calcium and diacylglycerol (DAG). The new PKCs (nPKC) include the isozymesε, η, δ, andθ, which are activated independently of calcium but in dependence of DAG. The atypical PKCs (aPKC) τ and ζ are activated independently of calcium and DAG. However, the role of PKCs in several important biological processes remains to be elucidated.

In a hyperglycaemic state not only hyperglycaemia per se, but also excessive neurohumoral stimulation are held responsible for the activation of the PKC isozymes [8,9]. In cardiac tissue, many of these neurohumoral peptides like angiotensin II, endothelin-1 or noradrenalin, which promote vasoconstriction and oxidative stress, bind to receptors that are coupled to the G_q_-protein Gα_11_. Interestingly the blockage of the renin-angiotensin-system (RAS) revealed a significant benefit in controlled randomised studies in a diabetic population [10,11]. These clinical observations are supported by animal models in which the blockage of the RAS showed a significantly diminished progression of atherosclerosis [12,13].

Based on these results we generated the hypothesis that a signalling pathway involving the G_q_-protein Gα_11 _may substantially mediate the pattern of PKC isoform expression in diabetes and therefore potentially atherogenesis.

To gain further insight in the complex pathological pathways, the present study analyzed the role of Gα_11 _on levels of expression of specific PKC isozymes distribution in the coronary vessels in an early diabetic environment.

## Methods

### Induction and verification of experimental induced STZ-diabetes

Investigations were carried out with Gα_11 _knockout mice and wildtype mice of the same line (C57/Bl6). Generation of the knockout mice was described previously [14]. C57BL6 wild type mice were obtained from Charles River Laboratories (Sulzfeld, Germany). The experimental design was approved by proper authorities and controlled by the Animal Welfare Officer of the University of Cologne (certificate AZ 50.203.2-K47,28/04). Mice 8 weeks of age were injected intraperitoneally with a single dose of either STZ in 0.1 mol/l citrate buffer, pH 4.5 (130 mg/kg), or citrate buffer. 24 hours later, blood glucose levels were determined using a Glucometer (GlucoMen Glycó) and Glucostix (GlucoMen Sensor, A. Menarini Diagnostics) to establish induction of diabetes. Afterwards the blood glucose levels and body weight of mice were monitored weekly. Successful induction of diabetes was defined by a constant blood glucose >300 mg/dl over 8 weeks. Throughout the study, animals were housed at 20-22°C with fixed 12 h day/night cycle and given free access to food and water.

### Sample Collection and Tissue Preparation

After 8 weeks of the diabetic status, animals were killed by cervical dislocation. Thoracic cavities were opened and hearts were removed and immersion-fixed using a fixative containing 4% paraformaldehyde and 0.2% picric acid in 0.1 M phosphate buffer salt (PBS), pH 7.4 for 24 h at 4°C. After washing in 0,1 M PBS, pH 7,4, 24 h, at 4°C, specimen were cryoprotected with 20% saccharose in 0,1 M PBS, pH 7,4, 48 h, at 4°C. Specimen were embedded in tissue-tek and quick-frozen in fluid nitrate. Specimens were cryosectioned with a thickness of 7 μm in consecutive sections. The hearts from 6 normal glycemic mice and 6 diabetic mice were presently analyzed in each group.

### Reagents and Antibodies

The rabbit anti-proteinkinase C δ-monoclonal antibody, the rabbit anti-protein kinase C βII-monoclonal antibody and the rabbit anti-protein kinase C ε-monoclonal antibody was purchased from Sigma Immunochemicals, St. Louis, MO, USA. The rabbit anti-protein kinase C ζ-monoclonal antibody and the rabbit anti-protein kinase C α-monoclonal antibody and were purchased from Cell Signaling Technology, Inc., Boston, MA, USA. All other reagents were of analytical grade or the best grade commercially available.

### Immunohistochemistry

In consecutive sections, Avidin-Biotin-Peroxidase Complex Method was performed for the immunohistochemical incubations (Elite ABC Kit, Vector Labor, Peterborough, UK). Endogenous peroxidases were inhibited with 0.3% H_2_O_2_. To block non-specific bindings, we treated sections with 1% bovine serum albumin (BSA) + 10% normal goat serum (NGS). Thereafter, sections were incubated for 24 hrs at 4°C with primary monoclonal antibodies against PKC isoforms followed by incubation with biotin-conjugated goat anti-rabbit IgG (1:500) and biotinylated anti-mouse IgG (1:500), respectively. The sections were then treated with avidin-biotinperoxidase complex (1:100) for 1 hr. The reaction was visualized with 0.05% 3,39-diaminobenzidine tetrahydrochloride (Sigma) in 0.05 M Tris-HCl buffer, pH 7.6, containing 0.01% H_2_O_2 _and 0.01% nickel ammonium sulphate. Incubations without the primary or secondary antisera and pre-absorption were carried out as negative controls.

### Quantification of Immunohistochemistry

The densitometric staining intensities of the PKC isoforms in treated and in control sections were measured by image analysis of grey values of immunostaining. The background grey value was measured from four selected regions at a cell free area. The blood vessels grey values were measured from four selected areas of the across sectioned blood vessels wall. Immunostaining intensity was presented as the mean of measured blood vessels grey value minus mean of measured background grey value. For staining intensity detection a Zeiss Axiophot microscope coupled to a 3-chip CCD-camera was used and the analysis was performed using the Optimas 6.00 image analysis program (Imaging Technology Inc., San Diego, CA, USA).

### Statistical analysis

Differences among groups were compared with student's t-test for unpaired observations. The data shown are means ± ± SE. The level of significance was set to P <0.05. All P values reported were based on two-sided tests.

## Results

### Induction of STZ-induced experimental diabetes

At the beginning of the study the animals showed no significant difference in the blood glucose levels. For those animals injected with citrate buffer the blood glucose level remained the same for the observed period of 8 weeks. Animals injected with STZ 130 mg/kg showed a significant increase to 491 ± 91 mg/dl in the wildtype group (P < 0,001) and to 432 ± 107 mg/dl in the Gα_11 _knockout group (P < 0,001) at the end of the in vivo study (Table 1). Another typical sign of type 1 diabetes beside the catabolic state was an observed polyuria and ketonuria. But the animals fed normally and moved around in their cages freely.

**Table 1:** Bodyweight and blood glucose levels of the animals at the end of the in vivo study.

	Wildtype Diabetes	Wildtype Control	Gα_11 _Knockout Diabetes	Gα_11 _Knockout Control
	n = 14	n = 10	n = 13	n = 13
Bodyweight (g)	20.5 ± 2.6 *	31.6 ± 2.5	25.2 ± 3.3 *	32.0 ± 5.1

Blood glucose (mg/dl)	491 ± 91 *	103 ± 12	432 ± 107 *	109 ± 9

Data are means ± plusorminus SEM.

* p < 0,05 vs. Control

### Coronary PKC expression in wildtype and Gα_11 _knockout animals under normoglycaemic conditions

**Figure 1:**
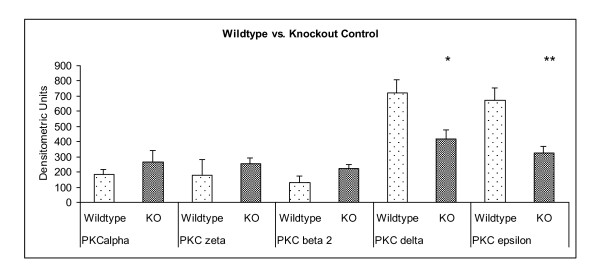
**Immunostaining of PKC isozymes in control Gα_11 _knockout animals compared to age matched control wildtype animals (Gα_11 _knockout n = 6, wildtype n = 6)**. The nPKC isozymes δ and ε showed a diminished expression in Gα_11 _knockout animals. KO = Knockout animals. * = p < 0.05 vs. Control. ** = p < 0.01 vs. Control.

**Figure 2:**
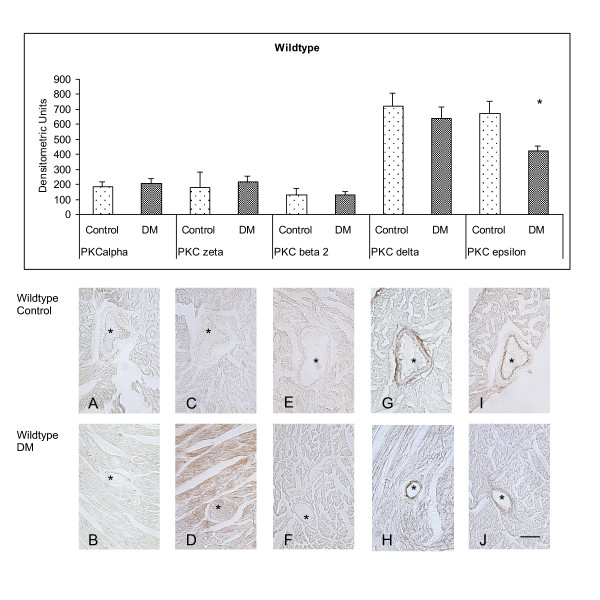
**Immunostaining of PKC isozymes in Wildtype animals after 8 weeks of experimental diabetes compared to age matched control animals (control n = 6, diabetes n = 6)**. PKC ε showed a diminished expression upon the induction of diabetes in wildtype animals. DM = Diabetes mellitus. * = p < 0.05 vs. control. bar = 60 μm.

**Figure 3:**
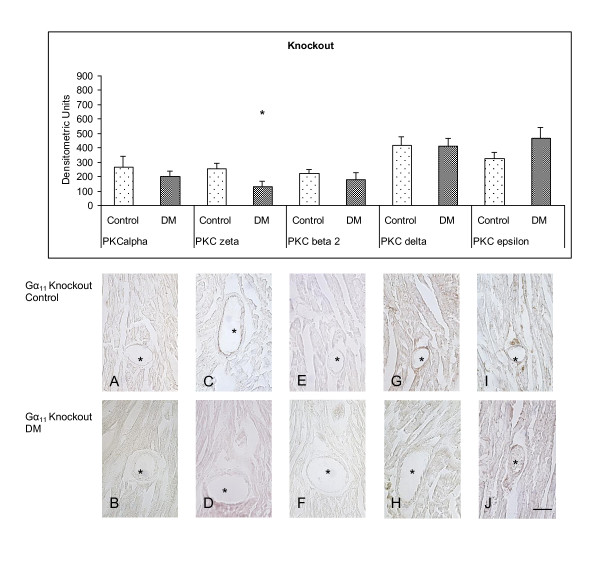
**Immunostaining of PKC isozymes in Gα_11 _knockout animals after 8 weeks of experimental diabetes compared to age matched control animals (control n = 6, diabetes n = 6)**. PKC ζ showed a decreased expression upon induction of diabetes in Gα_11 _knockout animals. DM = Diabetes mellitus. * = p < 0.05 vs. control. bar = 60 μm.

To evaluate the influence of the g-protein Gα_11 _on the coronary expression of the PKC isoforms α, βII, δ, ε and ζ, which are known to take crucial part in the pathogenesis of atherosclerosis, we compared wildtype control animals (WT) and Gα_11 _knockout control animals (KO) (Figure 1). Conventional PKC isoforms α (PKC α [densitometric Units] WT: 185.02 ± 32.37; KO: 267.45 ± 73.54; p = 0.28) (Figure 2A vs. Figure 3A) and βII (PKC βII; WT: 131.75 ± 41.62; KO: 222.18 ± 27.04; p = 0.098) (Figure 2E vs. Figure 3E) were equally expressed in wildtype and knockout animals. The analysis of the nPKC isoforms ε and δ both showed a significant attenuation of the expression in coronary vessels in Gα_11 _knockout animals (PKC δ [densitometric Units] WT: 719.84 ± 89.32; KO: 420.00 ± 58.38; p = 0.018) (Figure 2G vs. Figure 3G) (PKC ε; WT: 671.65 ± 81.3; KO: 327.15 ± 43.64; p = 0.0035) (Figure 2I vs. Figure 3I). The atypical PKC isoform ζ (PKCζ; WT: 180.48 ± 99.75; KO: 253.11 ± 38.45; p = 0.48) in turn was similarly expressed in the coronary vessels of wildtype and Gα_11 _knockout animals (Figure 2C vs. Figure 3C).

### Coronary PKC expression after 8 weeks of hyperglycaemia in wildtype and Gα_11 _knockout animals

After 8 weeks of diabetes the animals were sacrificed and the coronary vessels were assessed for PKC isoform α, βII, δ, ε and ζ expression. First we analyzed the expression of the conventional PKC isoforms (cPKC) α and βII in wildtype animals after 8 weeks of diabetes compared to age matched controls (Figure 2). We detected a low level of PKC α in diabetic animals (DM) and controls (C), which was equivalent (PKC α [densitometric Units] C: 185.02 ± ± 32.37; DM: 203.23 ± ± 37.62; p = 0.72) (Figure 2A vs Figure 2B). PKC βII in the coronary vessels showed only a faint expression with no alteration in the hyperglycaemic state (PKC βII [densitometric Units] C: 131.75 ± ± 41.62; DM: 132.42 ± ± 20.41; p = 0.99) (Figure 2E vs Figure 2F). We proceeded with examinations of the new PKC isoforms (nPKC) ε and δ. 8 weeks of diabetes did not change the high level of expression of PKC δ in coronary vessels of wildtype animals (PKC δ [densitometric Units] C: 719.84 ± ± 89.32; DM: 640.82 ± ± 76.3; p = 0.52) (Figure 2G vs Figure 2H) in contrast to the likewise high level of expression of PKC ε that was attenuated after 8 weeks of hyperglycaemia (PKC ε [densitometric Units] C: 671.65 ± ± 81.3; DM: 421.99 ± ± 35.86; p = 0.013) (Figure 2I vs Figure 2J). Next we studied the expression of the atypical PKC isoform (aPKC) ζ. This isoform did not show any significant alteration after 8 weeks of diabetes in wildtype animals (PKC ζ [densitometric Units] C: 180.48 ± ± 99.75; DM: 217.94 ± ± 39.17; p = 0.7) (Figure 2C vs Figure 2D).

In Gα_11 _knockout animals the same examinations were performed comparing the expression of the PKC isoforms α, βII, δ, ε and ζ in coronary vessels after 8 weeks of hyperglycaemia to age matched controls (Figure 3). Again the cPKC isoforms did not show any significant alterations (PKC α [densitometric Units] C: 267.45 ± ± 73.54; DM: 202.62 ± ± 36.63; p = 0.43) (Figure 3A vs Figure 3B) (PKC βII; C: 222.18 ± ± 27.04; DM: 180.28 ± ± 45.17; p = 0,43) (Figure 3E vs Figure 3F). PKC δ showed a lower level of expression in the coronary vessels of Gα_11 _knockout animals but again like in wildtype animals there was no change of expression detectable after 8 weeks of hyperglycaemia (PKC δ [densitometric Units] C: 420.00 ± ± 58.38; DM: 412.68 ± ± 52.04; p = 0.93) (Figure 3G vs Figure 3H). The second nPKC isoform analyzed in this study showed a different pattern of expression in G_11 _knockout animals than in wildtype animals after 8 weeks of diabetes. In Gα_11 _knockout animals the expression of PKC ε was not altered in the coronary vessels after the diabetic period of time (PKC ε [densitometric Units] C: 327.15 ± ± 43.64; DM: 464.1 ± ±76.79; p = 0.13) (Figure 3I vs Figure 3J). Again we proceeded with examination of the aPKC isoform ζ. However PKC ζ demonstrated a significant decrease in the expression in coronary vessels of Gα_11 _knockout mice after 8 weeks of diabetes (PKC ζ [densitometric Units] C: 253.11 ± ± 38.45; DM: 132.16 ± ± 33.8; p = 0.042) (Figure 3C vs Figure 3D) in contrast to wildtype animals.

## Discussion

Diabetes mellitus is a crucial risk factor for developing atherosclerosis. Common agreement exists on the role of excessive neurohumoral stimulation in the pathogenesis of atherosclerotic plaque formation in diabetes, involving angiotensin II and endothelin-1. These hormones bind to Gq-coupled receptors and may mediate their signal by differential regulation of specific PKC isoforms. Large clinical trials provided evidence of a lower incidence of ischemic heart disease when patients were treated with ACE inhibitors or AT1-receptor blockers compared to other antihypertensive drugs independent of blood pressure control [10,11]. These clinical observations are supported by animal models, in which the blockage of the renin angiotensin system (RAS) showed a significantly diminished progression of atherosclerosis [12,13]. The goal of the present study therefore was to gain further insight into the distribution of PKC isozymes in the coronary vessels in a model of an early diabetes mellitus and to define the role of the g-protein Gα_11 _on expression and localization of these kinases.

### Early diabetes shows no specific regulation of the conventional PKC isoforms α and βII

The conventional PKC isozymes α and βII have been shown to take part in different steps of atherogenensis. PKC α appears to be involved in O_2_^- ^mediated oxidation of LDL serving as a chemotactant for monocytes and may increase phospholipase A2 activation and monocyte platelet endothelial cell adhesion molecule (PECAM-1) expression, resulting in endothelial adhesion. PKC α has further been shown to stimulate VSMC for translocation and vasoconstriction [15] and to increase MMP-2 expression leading to plaque instability [16]. PKC βII appears to be involved in the expression of vascular cell adhesion molecule (VCAM) and inter-cellular adhesion molecule (ICAM) leading to monocyte recruitment and adhesion [17]. In our studies the levels of expression and location of conventional PKC isozymes α and βII were unchanged. Hence, in our present study there appears to be no specific regulation of these PKC isoforms in the coronary vessels in early diabetes.

### The atypical PKC isoform ζ is reduced in early diabetes after deletion of Gα_11_

Sorescu and coworkers showed that PKC ζ can activate the NADPH oxidase supporting the pro-oxidant environment of the atherosclerotic lesion by increasing reactive oxygen species production, thus leading to apoptosis and plaque instability [1]. In addition, the secretion of MMPs can be increased up to 13-fold through PKC ζ activity [18] promoting the degradation of extracellular matrix proteins. This pathomechanism has been linked to an increased risk of plaque rupture. In our Gα_11 _knockout model protein densities of PKC ζ were significantly diminished after 8 weeks of hyperglycaemia. In light of the current knowledge about the pathogenic role of PKC ζ in atherosclerosis this reduction in protein densities may thus reflect a protective mechanism in response to the deletion of the g-protein Gα_11_.

### Expression of the new PKC isoforms δ and ε is under control of Gα_11_

Compared to wildtype controls, expression of the nPKC isozymes δ and ε, which are activated independently of calcium but in dependence of DAG, were diminished in Gα_11 _knockout animals. This indicates strongly that expression of these isozymes is at least in part regulated by a Gα_11_-dependent pathway. Ma and coworkers provided evidence that PKC δ mediates the accumulation of oxidative LDL (oxLDL) in macrophages [19] leading to the formation of foam cells as one of the major mechanisms in the genesis of atherosclerotic lesions. Furthermore, there is evidence in support of a proapoptotic function of PKC δ in different cell types including VSMC [20]. In the pathogenesis of atherosclerosis, apoptosis of VSMCs is a central process leading to a marked thinning of the fibrous cap, the loss of collagen and matrix, accumulation of cell debris and intimal inflammation [21]. Following this line of evidence, the lower level of expression of the nPKC δ that we observed in nondiabetic Gα_11 _knockout mice compared to wildtype controls would target several processes that are crucial for atherosclerotic plaque formation. Hence, the Gα_11 _-dependent reduction in PKC δ expression could provide a protective mechanism in atherosclerotic heart disease. This view is supported by the persistent low expression of this isozyme in Gα_11 _knockout mice even after induction of diabetes, one of the primary risk factors for atherosclerosis.

The role of PKC ε in coronary vessels is not yet fully understood but evidence accumulates for an antiproliferative and antiapoptotic effect on VSMCs. Among others, this was nicely demonstrated by Sasaguri and coworkers showing that PKC ε suppresses proliferation of VSMCs by inhibition of the cell cycle at the late G1 phase [22]. In addition activation of PKC ε has been shown to protect against apoptosis through inhibition of the tumor necrosis factor TNFα and activation of transcriptional factor NF-κB via phosphorylation of IκB kinase [23-25]. Recently, Min and coworkers provided evidence for activation of the PKC/NF-κB pathway in neonatal rat cardiomyocytes exposed to high glucose further supporting the importance of this pathway in the diabetic heart [26].

It seems that PKC ε is further linked to raf-1 kinase activation and the mitogen-activated protein kinase pathway and it has been shown that this isoform mediates the effect of angiotensin II in the proximal tubule of the kidney [27]. In our present study protein levels of PKC ε were reduced in coronary vessels from wildtype mice 8 weeks after induction of diabetes. Similarly, the reduced expression of this isoform following hyperglycemia has previously been described in myocardial tissue [28,29] and a resulting susceptibility for postischemic damage has been postulated. If expression of the nPKCs δ and ε were under control of a Gα_11_-dependent signaling pathway we hypothesized that densities of these proteins would not be influenced by strong stimuli like hyperglycemia. The present results support this hypothesis, however, functional implications that would result from this permanent downregulation and possible mechanisms of compensation by other pathways are not yet fully understood. It is also to be considered that individual PKC isoforms may mutually regulate their activity as part of a concerted process involving a network of signalling cascades [30]. Further studies will be needed to address these functional aspects.

## Conclusion

In conclusion, the present study shows that expression of the nPKCs δ and ε in coronary vessels is under control of the g-protein Gα_11_. The reduced expression of PKC ζ that we observed in coronary arteries from Gα_11_-knockout mice compared to wildtype controls upon induction of diabetes could reduce apoptosis and promote plaque stability. These findings suggest a mechanism that may in part underlie the therapeutic benefit of RAS inhibition on cardiovascular endpoints in diabetic patients.

## Competing interests

The authors declare that they have no competing interests.

## Authors' contributions

DPH and YK carried out the immunohistochemistry and performed the statistical analysis. SG participated in the induction of the experimental Diabetes. KA, NW and SO participated in the design of the study. HR conceived of the study and participated in its design and coordination. All authors read and approved the final manuscript.
